# Epigenetic Regulation of *PLIN1* in Obese Women and its Relation to Lipolysis

**DOI:** 10.1038/s41598-017-09232-y

**Published:** 2017-08-31

**Authors:** Lucia Bialesova, Agné Kulyté, Paul Petrus, Indranil Sinha, Jurga Laurencikiene, Chunyan Zhao, Karin Dahlman Wright, Peter Arner, Ingrid Dahlman

**Affiliations:** 1 Department of Biosciences and Nutrition, Karolinska Institutet, S-141 83 Stockholm, Sweden; 2 Department of Medicine, Huddinge, Karolinska Institutet, S-141 86 Stockholm, Sweden

## Abstract

Increased adipocyte lipolysis links obesity to insulin resistance. The lipid droplet coating-protein Perilipin participates in regulation of lipolysis and is implicated in obesity. In the present study we investigate epigenetic regulation of the *PLIN1* gene by correlating *PLIN1* CpG methylation to gene expression and lipolysis, and functionally evaluating *PLIN1* promoter methylation. *PLIN1* CpG methylation in adipocytes and gene expression in white adipose tissue (WAT) was quantified in two cohorts by array. Basal lipolysis in WAT explants and adipocytes was quantified by measuring glycerol release. CpG-methylation of the *PLIN1* promoter in adipocytes from obese women was higher as compared to never-obese women. *PLIN1* promoter methylation was inversely correlated with *PLIN1* mRNA expression and the lipolytic activity. Human mesenchymal stem cells (hMSCs) differentiated *in vitro* into adipocytes and harboring methylated *PLIN1* promoter displayed decreased reporter gene activity as compared to hMSCs harboring unmethylated promoter. Treatment of hMSCs differentiated *in vitro* into adipocytes with a DNA methyltransferase inhibitor increased levels of *PLIN1* mRNA and protein. In conclusion, the *PLIN1* gene is epigenetically regulated and promoter methylation is inversely correlated with basal lipolysis in women suggesting that epigenetic regulation of *PLIN1* is important for increased adipocyte lipolysis in insulin resistance states.

## Introduction

Obesity is associated with adverse metabolic consequences including insulin resistance and development of type 2 diabetes^1^. One factor linking excess adipose tissue to metabolic disease is increased adipocyte lipolysis resulting in elevated circulating levels of non-esterified fatty acids (NEFA) in the circulation which in turn induce insulin resistance in other organs as reviewed^2, 3^.

During lipolysis, intracellular triacylglycerides (TAGs) undergo hydrolysis through the action of lipases. The regulation of lipolysis is complex. Hormones exert a tight control on lipolysis. In human the important hormones are catecholamines and heart-derived natriuretic peptides which stimulate, and insulin which inhibits, lipolysis^4^. Proteins covering lipid droplets in adipocytes also participate in regulation of lipolysis. Perilipin, encoded by the *PLIN1* gene, is the most extensively studied lipid droplet protein and inhibits basal lipolysis^5^. Lipolytic stimuli cause phosphorylation of Perilipin which facilitates hydrolysis of TAG by recruitment of hormone sensitive lipase to the lipid droplet^6, 7^.

Human adipocyte levels of Perilipin protein are inversely correlated with lipolytic rate^8^ supporting an important role of Perilipin in lipolytic regulation *in vivo*. Furthermore, obesity, which is associated with increased basal lipolysis, has repeatedly been shown to be associated with decreased levels of Perilipin protein in adipose tissue^8–10^. Adipose *PLIN1* mRNA has also been reported to be lower in obese as compared to lean subjects, although this has not been confirmed in all studies^9^. An intronic *PLIN1* gene*-*allele has been associated with higher basal lipolysis, as well as with reduced *PLIN1* content in adipocytes^8, 10, 11^, but the results are preliminary and need to be replicated in additional larger cohorts as discussed^12^. In addition, the transcription factors PPARG, NFkappaB, and LXRA control *PLIN1* gene transcription^13–15^. Beyond these transcriptional and possible genetic effects, the *in vivo* control of *PLIN1* levels is poorly defined. Adipocyte gene transcription is modulated by epigenetic mechanisms. Recently we reported dysregulated CpG-methylation of lipolytic genes as a major feature of the adipocyte epigenetic signature from obese woman; *PLIN1*, however, did not display significant differences in DNA-methylation in analysis of the global DNA methylome comparing obese and never-obese women^16^. Considering the importance of *PLIN1* for lipolysis, in the present study we used a candidate gene approach to address epigenetic regulation of the *PLIN1* gene in relation to lipolysis. We performed a comprehensive descriptive analysis of CpG methylation in relation to obesity and lipolysis *in vivo*, made a functional evaluation of CpG-methylation in the *PLIN1* promoter, and demonstrate that global demethylation increases levels of *PLIN1* mRNA and Perilipin protein.

## Results

### Clinical data

Clinical data for explorative and validation cohorts are presented in Table 1. Expected differences between obese and never obese women were observed. Thus, the obese women displayed a higher basal lipolysis in white adipose tissue (WAT) explants and in isolated adipocytes, which was accompanied by higher NEFA in the general circulation as compared to never-obese women. There was no significant difference in age between groups.

**Table 1:** Clinical characteristics of subjects.

	Explorative cohort			Validation cohort		
	Never-obese	Obese	*P* ^1^	Never obese	Obese	*P* ^1^
n	14	15		38	31	
Age (years)	45 ± 11	46 ± 11	0.93	40 ± 14	42 ± 11	0.65
Weight (kg)	69 ± 7	115 ± 11	5.1 × 10^−6^	66 ± 8	103 ± 20	1.0 × 10^−12^
BMI (kg/m^2^)	25.2 ± 2.5	41.4 ± 4.5	5.1 × 10^−6^	23.5 ± 1.9	39.9 ± 6.3	1.0 × 10^−11^
Waist to hip ratio	0.85 ± 0.06	0.98 ± 0.06	8.4 × 10^−5^	0.86 ± 0.07	0.97 ± 0.05	0.05
Systolic blood pressure (mmHg)	123 ± 19	138 ± 22	0.052	123 ± 17	128 ± 12	0.10
Diastolic blood pressure (mmHg)	74 ± 6	85 ± 9	6.0 × 10^−4^	74 ± 7	80 ± 7	0.0005
P-Glucose (mmol/L)	5.1 ± 0.4	5.7 ± 1.2	0.069	4.8 ± 0.5	5.1 ± 0.5	0.0014
P-Insulin (mU/l)	4.6 ± 2.3	16.0 ± 10.3	1.1 × 10^−4^	5.1 ± 2.5	13.9 ± 8.6	5.8 × 10^−8^
P-Cholesterol (mmol/l)	4.7 ± 1.0	4.9 ± 0.7	0.57	4.7 ± 0.9	5.0 ± 1.0	0.34
P-NEFA (mmol/l)	0.57 ± 0.17	0.83 ± 0.16	2.4 × 10^−4^	0.64 ± 0.18	0.78 ± 0.27	0.02
Basal lipolysis in isolated fat cells (micromoles of glycerol/2 h/10^7^adipocytes)^2^	1.34 ± 1.18	3.18 ± 1.47	0.0012	3.44 ± 2.24	8.67 ± 7.34	0.0004
Basal lipolysis in adipose tissue (micromoles of glycerol/2 h/10^7^ adipocytes)^3^	1.12 ± 0.64	3.43 ± 0.91	1.0 × 10^−4^	2.96 ± 1.52	5.59 ± 2.77	2.3 × 10^–5^
Average adipocyte volume, picolitres	442 ± 169	994 ± 184	5.5 + 10^−8^	471 ± 169	907 ± 194	1.0 × 10^−10^

^1^Comparison of never-obese and obese group with unpaired t-test or Kruskal-Wallis test as indicated in the Statistical analysis section. Values are mean ± SD.

^2^Data available for 14 never-obese and 14 obese women in the explorative cohort, and for 37 never-obese and 31 obese women in the validation cohort.

^3^Data available for 5 never-obese and 12 obese women in the explorative cohort, and for 32 never-obese and 29 obese women in the validation cohort.

### *PLIN1* in relation to obesity

**Figure 1:**
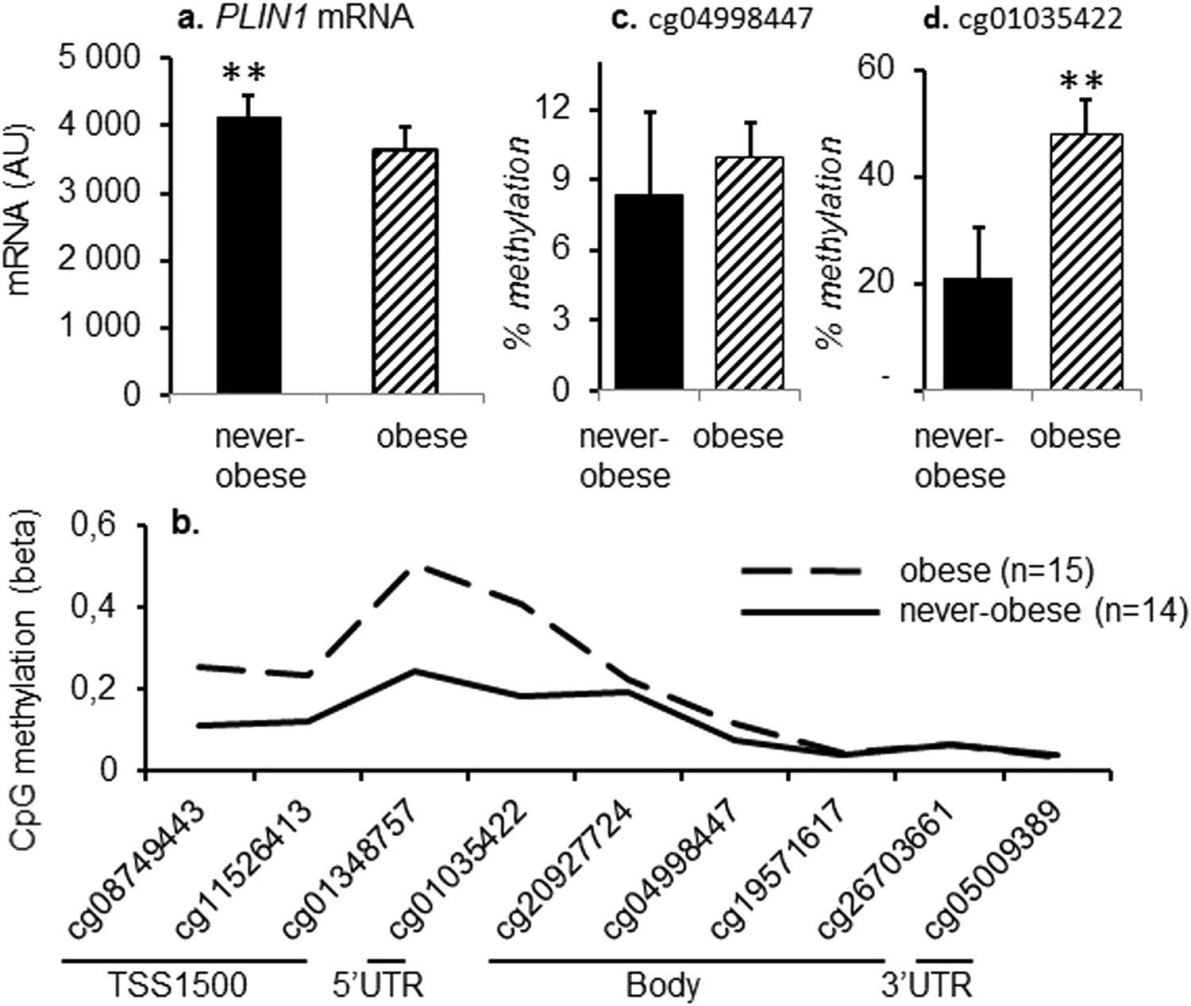
*PLIN1* mRNA levels and CpG-methylation in obese and never-obese women from the explorative cohort. (**a**) *PLIN1* mRNA in WAT of obese (n = 9) and never-obese (n = 9) women. *PLIN1* mRNA quantification was performed with microarray. (**b**) Average methylation of several CpG-sites in the *PLIN1* gene assessed by microarray in adipocytes from obese (n = 15) and never-obese (n = 14) women. (**c**) Methylation of cg04998447 in adipocytes from obese (n = 5) and never-obese (n = 9) women. (**d**) Methylation of cg01035422 in adipocytes from obese (n = 5) and never-obese (n = 9) women. Quantification of CpG methylation in c. and d. was performed with Pyrosequencing. Values are mean ± SD. **P < 0.01.

We confirmed that obese women displayed lower expression of *PLIN1* mRNA in subcutaneous adipose tissue (*P* = 0.0058) (Fig. 1a), whereas CpG-methylation of the *PLIN1* gene in adipocytes was higher (Fig. 1b), as compared to never-obese women. Differential methylation was most pronounced in the promoter and 5′ region of the gene, and CpG-methylation of adipocyte DNA, quantified as beta-value, was higher in obese as compared to never-obese women of both the explorative and validation cohorts (Table 2). CpG methylation of the *PLIN1* gene was not dependent on age in the explorative cohort, whereas a positive correlation was observed in the validation cohort (Table 2). Age did not impact the correlation between CpG-methylation and any other investigated phenotype such as BMI or lipolysis. We validated differentially methylated sites (DMS) by Pyrosequencing in a subset of samples from the explorative cohort (5 obese and 9 never-obese women) and were able to analyze assays for two out of three tested CpG-sites. Results were directionally consistent for cg04998447 (Fig. 1c), and directionally consistent as well as significant for cg01035422 (*P* 0.0041) (Fig. 1d). The assay for cg08749443 failed in the clinical cohort.

**Table 2:** DMS in *PLIN1* in relation to obesity and age.

Probe	Position	Gene	Relation to CpG	Obesity status			Age	
		region	Island	Never-obese	Obese	*P* ^1^	Std beta	*P* ^4^
**Explorative cohort**								
cg08749443	90224098	TSS1500	Open sea	0.114 ± 0.045	0.255 ± 0.113	1.1 × 10^−4^	−0.119	0.54
cg11526413^2^	90223712	TSS1500	Open sea	0.122 ± 0.056	0.233 ± 0.100	2.7 × 10^−4^	−0.107	0.58
cg01348757^2^	90223301	TSS1500	Open sea	0.244 ± 0.095	0.505 ± 0.142	1.1 × 10^−4^	0.046	0.81
cg01035422	90222555	5′UTR	Open sea	0.181 ± 0.067	0.407 ± 0.122	2.5 × 10^–5^	−0.045	0.82
cg23205660^3^	90212699	Body	S_Shelf					
cg20927724	90209326	Body	S_Shore	0.200 ± 0.030	0.226 ± 0.047	0.012	0.044	0.92
cg04998447	90209223	Body	Island	0.078 ± 0.018	0.117 ± 0.027	1.1 × 10^−4^	0.078	0.68
cg19571617	90209190	Body	Island	0.038 ± 0.009	0.046 ± 0.015	0.16	−0.007	0.97
cg26703661	90208915	Body	Island	0.064 ± 0.009	0.065 ± 0.021	0.62	0.08	0.68
cg05009389	90208810	3′UTR	Island	0.041 ± 0.017	0.037 ± 0.008	0.68	0.052	0.79
cg26585724^3^	90208739	3′UTR	N_Shore					
**Validation cohort**								
cg08749443	90224098	TSS1500	Open sea	0.254 ± 0.105	0.331 ± 0.069	0.0008	0.318	0.0077
cg11526413^2^	90223712	TSS1500	Open sea	0.268 ± 0.120	0.331 ± 0.084	0.0078	0.279	0.020
cg01348757^2^	90223301	TSS1500	Open sea	0.421 ± 0.177	0.517 ± 0.091	0.004	0.384	0.0011
cg01035422	90222555	5′UTR	Open sea	0.379 ± 0.135	0.483 ± 0.078	0.0004	0.332	0.0054
cg23205660^3^	90212699	Body	S_Shelf					
cg20927724	90209326	Body	S_Shore	0.212 ± 0.040	0.229 ± 0.032	0.066	0.380	0.0013
cg04998447	90209223	Body	Island	0.089 ± 0.016	0.101 ± 0.018	0.021	0.364	0.0021
cg19571617	90209190	Body	Island	0.053 ± 0.010	0.058 ± 0.009	0.014	0.112	0.359
cg26703661	90208915	Body	Island	0.048 ± 0.012	0.044 ± 0.007	0.24	−0.017	0.89
cg05009389	90208810	3′UTR	Island	0.035 ± 0.009	0.032 ± 0.006	0.15	−0.0074	0.95
cg26585724^3^	90208739	3′UTR	N_Shore					

^1^Comparison of never-obese and obese group with Kruskal-Wallis test. Values are mean ± SD.

^2^SNP within 10 bps of interrogated CpG-site.

^3^SNP with MAF >10% in probes.

^4^Relationship between age and CpG-methylation was assessed by simple regression.

### *PLIN1* in relation to lipolysis

**Figure 2:**
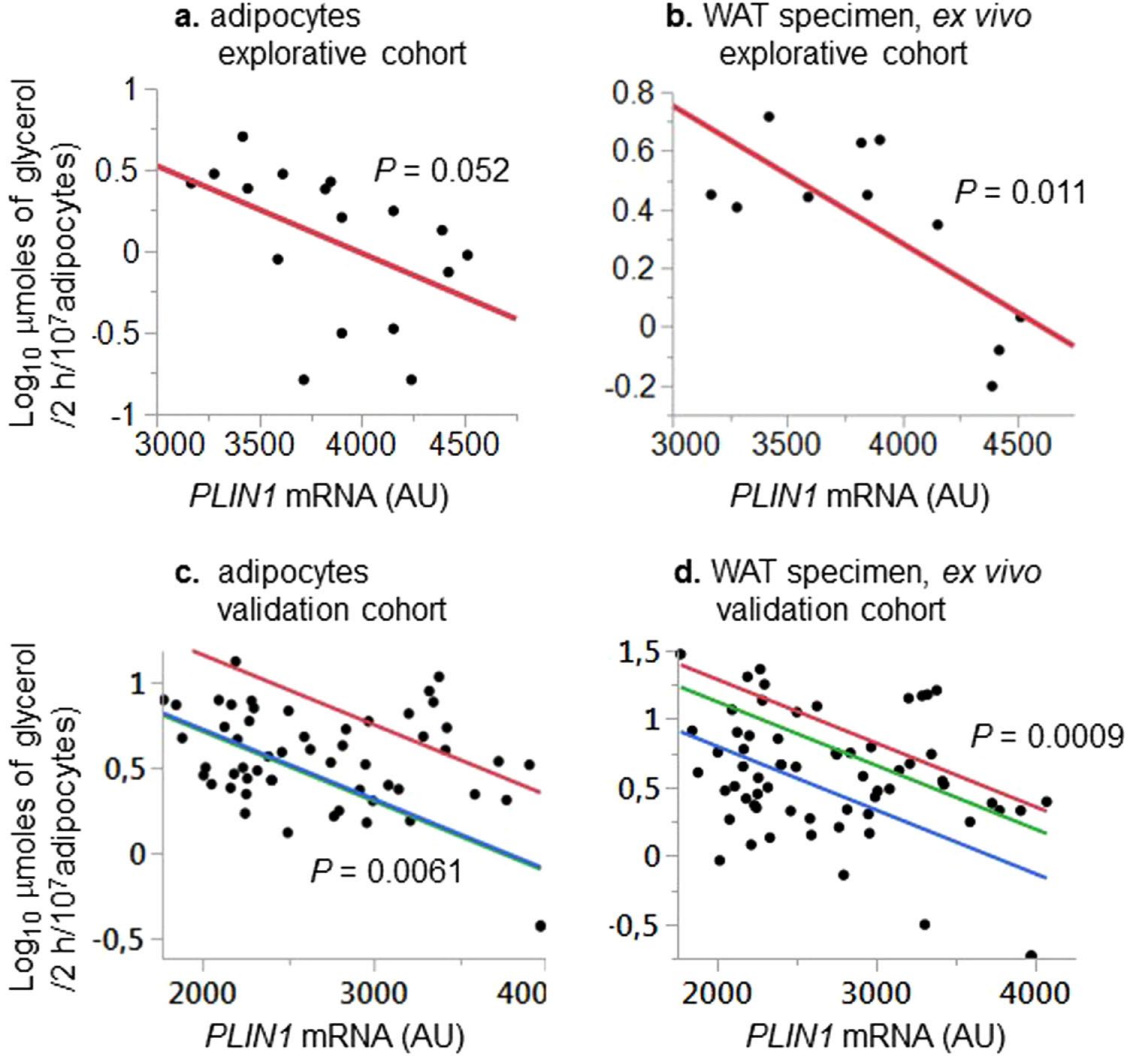
*PLIN1* mRNA in relation to adipose basal lipolysis. (**a**) Basal lipolysis quantified in isolated subcutaneous adipocytes from women in the explorative cohort (n=17). (**b**) Basal lipolysis quantified *ex vivo* in adipose tissue explants obtained by needle aspiration from women in the explorative cohort (﻿n=11). (**c**) Basal lipolysis quantified in isolated subcutaneous adipocytes from women in the validation cohort (n=56). (**d**) Basal lipolysis quantified *ex vivo* in adipose tissue explants obtained by needle aspiration from women in the validation cohort ﻿(n=49). See methods section for details about assays for quantifications of lipolysis and *PLIN1* mRNA. Results were analyzed by simple (Fig. 2a and b) or multiple (Fig. 2c and d) regression adjusting for array batch.

We further studied adipocyte CpG-methylation of *PLIN1* in relation to lipolysis in more detail. There were inverse correlations between *PLIN1* mRNA and basal lipolysis in isolated adipocytes (Fig. 2a,c) and WAT explants (Fig. 2b,d) in both the explorative and validation cohorts. Methylation of all examined CpG-sites in the *PLIN1* gene promoter and 5′ region in adipocytes was inversely related to *PLIN1* mRNA, as well as WAT *ex vivo* and adipocyte basal lipolysis (Table 3). By contrast, only one of five examined CpG-sites in the *PLIN1* gene body and 3′ UTR was nominally associated with these phenotypes.

**Table 3:** Relationship between methylation of CpG-sites in the *PLIN1* gene, *PLIN1* mRNA levels and lipolysis^1^.

*PLIN1* CpG methylation^2^		*PLIN1* mRNA^3^		Basal lipolysis in adipocytes^4^		Basal lipolysis in WAT explants^4^	
Probe	Gene region	Std beta	*P*	Std beta	*P*	Std beta	*P*
**Detection cohort**							
cg08749443	TSS1500	−0.531	0.023	0.515	0.0051	0.437	0.079
cg11526413^5^	TSS1500	−0.649	0.0036	0.454	0.015	0.477	0.053
cg01348757^5^	TSS1500	−0.612	0.007	0.543	0.0028	0.612	0.0091
cg01035422	5′UTR	−0.592	0.0096	0.635	0.0003	0.625	0.0073
cg23205660^6^	Body						
cg20927724	Body	−0.368	0.133	0.47	0.012	0.238	0.357
cg04998447	Body	−0.471	0.048	0.503	0.0064	0.61	0.0093
cg19571617	Body	−0.415	0.087	0.342	0.074	0.427	0.087
cg26703661	Body	−0.045	0.859	0.146	0.457	0.249	0.335
cg05009389	3′UTR	0.181	0.472	0.239	0.221	0.013	0.96
cg26585724^6^	3′UTR						
**Validation cohort**							
cg08749443	TSS1500	−0.293	0.027	0.354	0.0024	0.432	0.0004
cg11526413^5^	TSS1500	−0.36	0.006	0.281	0.017	0.376	0.0022
cg01348757^5^	TSS1500	−0.315	0.017	0.256	0.032	0.385	0.0017
cg01035422	5′UTR	−0.318	0.016	0.317	0.0071	0.412	0.0007
cg23205660^6^	Body						
cg20927724	Body	−0.134	0.32	0.132	0.27	0.286	0.022
cg04998447	Body	−0.153	0.26	0.285	0.012	0.386	0.0016
cg19571617	Body	−0.018	0.9	0.223	0.061	0.278	0.026
cg26703661	Body	−0.042	0.76	0.018	0.88	0.002	0.98
cg05009389	3′UTR	−0.086	0.53	0.068	0.57	−0.0062	0.96
cg26585724^6^	3′UTR						

^1^Relationship between degree of CpG-methylation and quantitative phenotypes was assessed by simple regression. ^2^ *PLIN1* CpG methylation was analyzed in DNA extracted from adipocytes using the Infinium Human Methylation 450 (explorative cohort) or EPIC (validation cohort) arrays. ^3^ *PLIN1* mRNA in WAT was quantified by microarray. ^4^Log10 micromoles of glycerol/2 h/107 adipocytes. ^5^SNP within 10 bps of interrogated CpG-site. ^6^SNP with MAF > 10% in probes.

**Figure 3:**
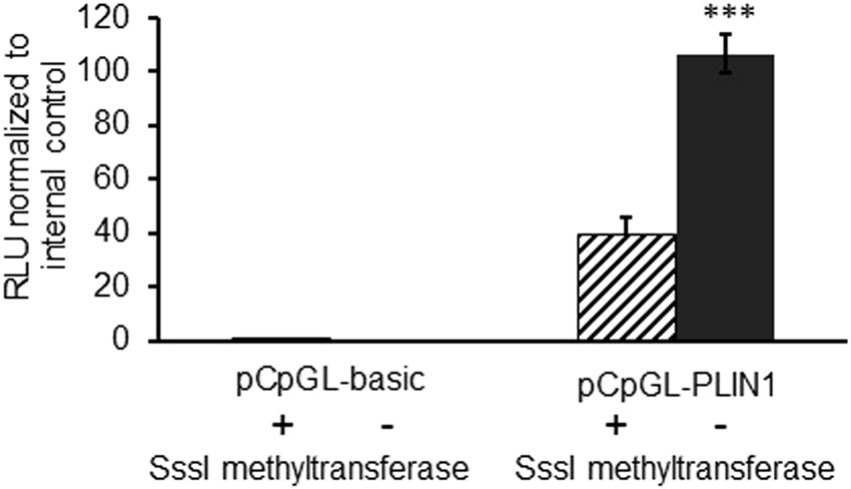
*PLIN1* promoter methylation inhibits promoter activity. *PLIN1* promoter activity is decreased after (hatched bar) versus without (black bar) DNA methylation by SssI methyltransferase. hMSCs were transfected with methylated and unmethylated pCpGL-*PLIN1* plasmid. As negative control, cells were transfected with empty vector, pCpGL-basic. Each sample was prepared in quadruplicates and the experiment was repeated three times. Y axis is the ratio between firefly and renilla luciferase. Renilla luciferase is expressed from a second plasmid as an internal control. RLU = Relative luciferase units. ***P < 0.001.

To assess whether CpG-methylation of *PLIN1* affects promoter activity, a luciferase reporter gene assay was used. hMSCs were transfected with a plasmid containing the *PLIN1* promoter region cloned into a CpG free backbone vector (pCpGL-PLIN). Cells transfected with methylated pCpGL-PLIN plasmid displayed a significant and marked decrease (>60%) of luciferase activity in comparison to those transfected with unmethylated pCpGL-PLIN plasmid (*P* < 0.001). The methylated and unmethylated control plasmid showed no difference in reporter gene activity (Fig. 3).

**Figure 4:**
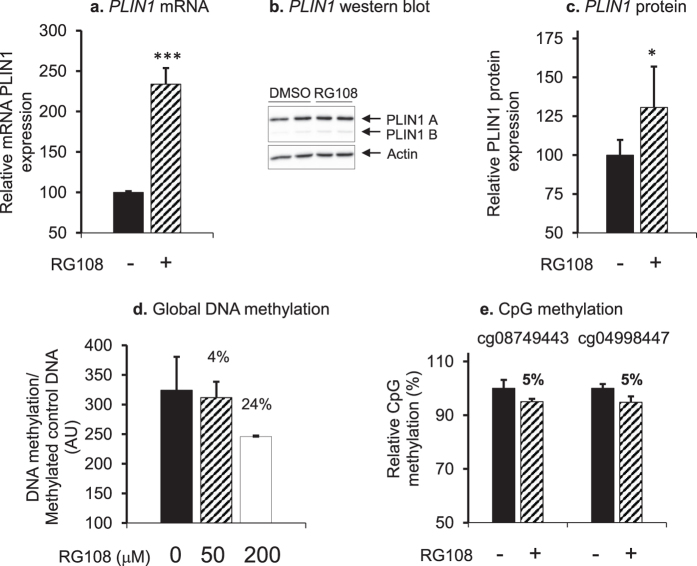
Global demethylation affects Perilipin levels in adipocytes. (**a**) ***PLIN1*** mRNA as determined by RT-qPCR and (**b,c**) Perilipin protein as determined by Western blot were increased after treating hMSCs with DNA methyltransferase inhibitor RG108 (50 µM) (hatched bars) as compared to vehicle (black bars). The experiment was repeated three times. Representative Western blots are shown. These blot pictures were cropped and the full-length blot pictures are presented in Supplementary Fig. S1. Results are presented as relative fold change ± SD vs. vehicle-treated cells. (**d**) Global DNA methylation in adipocytes was decreased after 24 h treatment with DNA methyltransferase inhibitor RG108 at a concentration of 50 µM (hatched bar) or 200 µM (white bar) compared to non-treated control cells (black bar). The experiment was repeated twice. (**e**) The methyltransferase inhibitor RG108 decreased methylation of specific CpG-sites in the *PLIN1* promoter in adipocytes. Methylation of cg08749443 and cg04998447 was determined by Pyrosequencing after adipocytes were treated with 50 µM RG108 (hatched bar) compared to non-treated control cells (black bar). The analysis was repeated twice, n > 3. ***P < 0.001, **P < 0.01, *P < 0.05.

To further evaluate epigenetic regulation of *PLIN1*, hMSCs differentiated *in vitro* to adipocytes were treated with 50 or 200 µM of DNA methyltransferase inhibitor RG108 for 24 h. The higher concentration of RG108 (200 µM) was determined to be cytotoxic (results not shown), therefore all subsequent experiments were performed using 50 µM RG108. As a consequence of RG108 treatment, levels of *PLIN1* mRNA (Fig. 4a) and Perilipin protein (Fig. 4b,c) were upregulated by about 140% and 30%, respectively. Demethylation activity of RG108 was confirmed by Global DNA methylation Imprint^®^ Methylated DNA Quantification Kit, and showed that treatment of adipocytes with RG108 decreased global DNA methylation by 4% and 24%, respectively (Fig. 4d). To confirm that RG108 demethylated *PLIN1*, two CpG sites in the promoter were selected for analysis by Pyrosequencing (cg08749443 and cg04998447). The results showed that 50 µM RG108 decreased methylation at both CpGs by 5% (Fig. 4e).

## Discussion

We herein report that *PLIN1*, a key regulator of basal lipolysis, is subject to functional regulation by epigenetic modifications. It is demonstrated that CpG methylation of the *PLIN1* gene promoter inhibits promoter activity *in vitro*, and that adipocyte promoter methylation is inversely correlated with *PLIN1* mRNA levels in clinical cohorts. Furthermore, a positive correlation between *PLIN1* CpG methylation and adipose basal lipolysis *ex vivo* was found. This indicates that epigenetic regulation is important for the regulation of lipolysis in human WAT.

Differential CpG methylation of *PLIN1* was not evident in our original published global methylome analysis of obese versus never-obese women. This is most likely due to the use of a stringent threshold to adjust for multiple testing^16^. However, the validation of DMS in the *PLIN1* promoter in the present study using two cohorts with array data, as well as an independent method (Pyrosequencing) clearly establishes the presence of differential methylation of *PLIN1* in obesity. Furthermore, there is a positive correlation between *PLIN1* CpG-methylation in intact WAT and BMI (see supplementary tables in Rönn T *et al*.^17^). The reported relationship is weaker than the one observed here, which might be due to the fact that we study isolated adipocytes and hereby avoid the confounding effects of other cell types in intact WAT. By contrast CpG-methylation of *PLIN1* has not been associated with BMI in reported epigenome-wide association studies on whole blood or leukocytes suggesting that the effect is specific to adipocytes or WAT where *PLIN1* influences lipolysis^18, 19^. We observed some correlation between CpG methylation of the *PLIN1* promoter and age in the validation, but not in the explorative cohort. This is in agreement with reported finding for WAT that, although the average methylation of CpG-sites covering the genome is positively correlated with age, only a minor proportion of individual CpG-sites display significant correlation between methylation and age^17^. Specifically, CpG-methylation of *PLIN1* did not correlate with age in the large cohorts of men and women studied by Rönn *et al*.

Increased lipolysis is implicated in insulin resistance, as well as in a number of other conditions including cachexia, hepatosteatosis, and cardiovascular disease^2, 20–22^. In addition, there is evidence for epigenetic dysregulation in these disease states^23–25^. Despite the importance of *PLIN1* for lipolytic regulation^5^, the *PLIN1* gene locus has not come out as a susceptibility locus in genome-wide association studies for e.g. obesity or insulin resistance^26–28^. The finding that CpG methylation controls the activity of the *PLIN1* promoter thus shed new light on the regulation of adipocyte lipolysis and potentially why lipolysis is altered in metabolic diseases. The degree of global DNA demethylation, 4–24%, reported here is in the same range as has been reported previously when treating non-dividing cells with a global methyltransferase inhibitor^29^. Future work is needed to define how CpG methylation interacts with other known regulators of lipolysis^4^. In the present study methylation of all examined CpG-sites in the *PLIN1* promoter were positively associated with obesity and lipolysis in the clinical cohorts. Transcriptional regulation of *PLIN1* is incompletely defined; the transcription factors PPARG, NFkappaB and LXRA have been shown to regulate *PLIN1* transcription^13–15^. The reported binding motifs for PPARG and LXRA do not contain any CpG-sites, nor the predicted binding site for NFkappaB. As regards the studied CpG-sites in the *PLIN1* promoter, cg08749443 overlaps a predicted binding site for Sp1, whereas the other CpG-sites show no overlap with predicted transcription factor binding sites according to AliBaba using the public Transfac database to predict transcription factor binding matrices (http://www.gene-regulation.com/pub/databases.html). The results, thus, do not permit us to draw any conclusion if methylation of some part(s) of the promoter is more important than others.

What might in turn regulate CpG-methylation of the *PLIN1* promoter? It is established that external factors such as diet and physical exercise influence the methylome^30–32^; however, in the few available human intervention studies that have the DNA methylome as one outcome neither physical exercise nor high-fat overfeeding are associated with differential CpG methylation of *PLIN1* ^30, 32^. Thus, it remain to establish if and which behavioral factors influence CpG-methylation of the *PLIN1* promoter. In addition, it is possible that metabolic factors such as fat mass (obesity) and enlarged fat cells are more important.

There are some limitations with the present study. We only examined women and one adipose region. Perilipin protein in WAT has been reported to be higher in men than in women^9^. Unfortunately, we do not have isolated adipocytes from men and therefore cannot investigate in clinical cohorts if epigenetic regulation of *PLIN1* is gender specific. It is possible that findings in men and fat cells from other depots such as visceral WAT are somewhat different although it is unlikely that such variations are of qualitative nature. Furthermore, investigations of visceral adipose tissue necessitate the use of general surgery procedures which by themselves alter WAT gene expression^33^.

In conclusion, the *PLIN1* gene is epigenetically regulated and promoter methylation is inversely correlated with basal lipolysis in women suggesting that epigenetic regulation of *PLIN1* is important for increased adipocyte lipolysis in insulin resistant states such as obesity.

## Methods

### Subjects

The subjects in this study have been described before^16^. Briefly, the discovery cohort comprised fifteen obese women (body mass index (BMI) >30 kg/m^2^) and fourteen never-obese healthy control women (BMI <30 kg/m^2^) who were recruited in association with planned visits to our surgical units for gastric by-pass surgery because of obesity and through local advertisement for the purpose of studying WAT factors regulating body weight. Clinical data are presented in Table 1. All 14 never-obese women were healthy. Three of the obese women had type 2 diabetes, out of which two were treated with diet and metformin, and one subject with diet alone. CpG methylation of examined CpG-sites did not differ significantly between metformin treated and other women. For six CpG-sites methylation beta-values in the metformin treated women were within the interval defined by the average + SD for the women not treated with metformin; for remaining three CpG-sites the methylation beta-value of 1≥ subject was outside this region, but not in a consistent direction. Overall, this suggests that metformin does not influence methylation of *PLIN1*. Nine of the obese individuals were treated for hypertension and one patient had stable multiple sclerosis and did not receive any drugs. The women undergoing gastric by-pass surgery participated in a trial on the effect of bariatric surgery (NCT01785134 at www.clinicaltrials.gov) and were investigated before surgery. The study was approved by the regional ethics board in Stockholm and written informed consent was obtained from each subject. The experiments conformed to the principles set out in the WMA Declaration of Helsinki and the Department of Health and Human Services Belmont Report.

Transcriptome analysis on WAT specimens was conducted for 18 of the above individuals (9 obese, and 9 never-obese). For the remaining subjects included in this study we did not have sufficient amount of WAT for transcriptome analysis.

For validation of *PLIN1* results we studied an independent group of women with a wide variation in BMI (n = 69, age 40 ± 12 years, BMI 30.9 + 9.3 kg/m^2^) who were examined in the same manner as the explorative cohort. Global transcriptome profiles was available on subcutaneous WAT from 57 of these women as reported^34^.

### Clinical examination

Participants were investigated at 8 AM after an overnight fast. All subjects had been weight stable (< ± 2 kg body weight change) during at least 6 months prior to investigation according to self-report Anthropometric measurements were performed followed by venous blood sampling. Plasma and serum were used for analysis of NEFA and other clinical chemistry variables as described^35^. An abdominal subcutaneous WAT biopsy was obtained by fine needle aspiration as described^36^.

### WAT handling

The adipose tissue was brought to the laboratory, rinsed repeatedly in saline and visual blood vessels and cell debris were removed. Adipose tissue specimen (about two grams) were divided into portions, one of which was subjected to collagenase treatment to obtain isolated adipocytes as previously reported^37^. The mean weight and volume of these cells were determined as previously described^38, 39^. 200 µl of packed isolated adipocytes and 300 mg unfractionated WAT pieces were frozen in liquid nitrogen and kept at −70 °C for subsequent DNA (cells) or RNA (tissue) preparation, whereas remaining tissue was used immediately for cell culture experiments.

### Lipolysis assay

Basal lipolytic activity was determined in adipose tissue explants as described^40^. In brief, pieces of adipose tissue (200 or 300 mg) were incubated for 2 h (100 mg/ml) at 37 °C with air as the gas phase in Krebs–Ringer phosphate buffer (pH 7.4) supplemented with glucose (8.6 mmol/l), ascorbic acid (0.1 mg/ml) and bovine serum albumin (20 mg/ml). Glycerol release into the medium was measured using a sensitive bioluminescence method and expressed as amount of glycerol release per 2 h and 10^7^ adipocytes. Adipocytes are the only adipose source of glycerol, which is an end product of lipolysis and only metabolized by adipose tissue to a minimal extent. Methodological experiment revealed that glycerol release in these type of experiments is linear with incubation time for at least 4 h. Cross-sectional and longitudinal studies have demonstrated that basal lipolytic activity is strongly and negatively related to *in vivo* insulin sensitivity.

Basal lipolysis in isolated adipocytes was investigated using the same protocol as described above in diluted adipocyte suspensions (2% v/v).

### Quantification of *PLIN1* CpG methylation by array

*PLIN1* CpG methylation in the explorative cohort was analyzed in DNA extracted from adipocytes in a previously reported dataset applying the Infinium Human Methylation 450 BeadChip assay (Illumina, San Diego, CA, USA)^16^. The beta value (β), which represents the ratio of intensities between methylated and unmethylated alleles, was used to quantify methylation at specific CpG loci. The β-values vary from 0 (no methylation) to 1 (100% methylation). Of 11 probes mapping to the *PLIN1* gene, we excluded two containing SNPs with MAF >10% within the probes according to Illumina annotation.

CpG-methylation in adipocytes from the validation cohort was analyzed by EPIC arrays. Briefly, 500 ng of genomic DNA was bisulfate converted with EZ-96 DNA Methylation kit (Zymo Research, Irvine, CA, USA) and genome wide DNA methylation analysis was performed using the Infinium Human Methylation EPIC BeadChip (Illumina, San Diego, CA, USA). The laboratory procedures were performed according to the manufacturer’s protocol. For analysis, visualization and extraction of methylation data, the GenomeStudio software version 2011.1 (Illumina Inc.) was used. The analysis reported here was limited to CpG-sites in the *PLIN1* gene region that should be confirmed from the explorative cohort. A comprehensive analysis of the full EPIC data is ongoing.

### Pyrosequencing assay

Five of the obese and nine of the never-obese women were used for validation of DMS by pyrosequencing; from remaining women we did not have any cells left. Genomic DNA was prepared from adipocytes using the QiAamp DNA Mini kit (Qiagen, Hilden, Germany). The DNA purity and quality was confirmed by A260/280 ratio >1.8 on a Nanodrop ND-1000 Spectrophotometer (Thermo Fisher Scientific Inc., Waltham, MA, US). The DNA concentration was measured by Qubit (Life technologies, Stockholm, Sweden).

Adipocyte genomic DNA (200 ng) was bisulfite converted according to the manufacturer’s instructions using the EZ DNA Methylation-Gold™ Kit (Zymo Research, Orange, CA, USA). Primers for PCR amplification and sequencing were designed using the PyroMark Assay Design 2.0 (Qiagen,). The following primers were used; for cg01035422 forward: TGTAAGGTAGGTGTTTTAGGATTTTAATA; biotinylated reverse: TAACCCTATTATCTCTCCCTCTCT and sequencing: GGTGTTTTAGGATTTTAATATTTAT. The nucleotide dispensing order was: TTYGGTTGAT CGTTATTTTA GTTTTATA; for cg04998447 forward: TTTGGGGAGTTGAGGGTT; biotinylated reverse: CCCCAACCTATATCCTCCT and sequencing: AGTTTTGGTTTGGTTTT. The nucleotide dispensing order was: GGGTTTTGYG TTTTTGATTT AT. Finally for cg08749443 biotinylated forward: TTTAGGAGAGTTTAGAGGGAAGATAGAAGT; reverse: AACCTAAATCCCTACTCTCACTTAATAA and sequencing: AAAAATAAAAAAAACAAATAAATAC. The dispensing order was: RAAAAAAAAA AAAAAATAAA ACTCTA. One µl bisulfite converted DNA was amplified using the PyroMark PCR kit (Qiagen) according to the manufacturer’s instructions applying annealing temperature of 58 °C. The entire PCR product was mixed with 4 µM sequencing primer and sepharose beads (GE Healthcare, Danderyd, Sweden) and bisulfite pyrosequenced using the PSQ 96 ID system (Qiagen) with PyroMark Gold Q96 reagents (Qiagen).

### Quantification of *PLIN1* expression

*PLIN1* mRNA in the clinical samples was quantified by microarray using Gene 1.1 ST arrays (Affymetrix, Santa Clara, CA, US) in 20 of the individuals in the explorative cohort, and by Gene 1.0 or 1.1 ST arrays in 57 of the women in the validation cohort; both groups formed parts of larger studies as described^16, 34^. For remaining subjects we did not have sufficient amount of WAT for transcriptome analysis.

Total RNA and DNA was extracted from cell culture samples using the AllPrep DNA/RNA Kit (Qiagen). Concentration and purity of nucleic acids was measured using a Nanodrop ND-1000 Spectrophotometer (Thermo Fisher Scientific, Lafayette, CO). cDNA synthesis was performed using iScript cDNA Synthesis kit (Bio-Rad Laboratories, Hercules, CA) together with random hexamer primers (Invitrogen, Carlsbad, CA). Assessment of *PLIN1* mRNA levels was performed using SYBR Green Mix (Bio-Rad Laboratories) and primers: *PLIN1* forward 5′-TGGAAACTGAGGAGAACAAG-3′ and reverse 5′-ATGTCACAGCCGAGATGG-3′. Expression was normalized to the internal reference gene *18S* forward 5′-TGACTCAACACGGGAAACC-3′ and reverse 5′-TCGCTCCACCAACTAAGAAC-3′ using the ΔΔCt method^41^.

### Construction of reporter vector

*PLIN1* promoter was PCR amplified using KAPA HotStart ReadyMix (Kapa Biosystems, Wilmington, MA) from human genomic DNA (Roche, Basel, Switzerland) using primers, forward 5′-TATTGGATCCGTACAGCCCAGCACATTCACAACT-3′ and reverse 5′-TATTAAGCTTGCCCCAGGACCCCAACAC-3′ (Sigma Aldrich, Dorset, UK) and cloned into the pCpGL-basic vector (kindly provided by prof. M. Rehli, Regensburg, Germany) via *BamH*I and *Hind*III (Thermo Scientific) sites, which yielded the pCpGL-PLIN vector. The correct insertion of a construct was controlled by sequencing. The cloned genomic sequence covered 1,731 base pairs upstream of the *PLIN1* transcription start site, and included all CpG-sites in the promoter region of *PLIN1* whose methylation status was assayed by microarray.

### *In vitro* methylation of plasmid DNA

Both plasmids, pCpGL-PLIN and pCpGL-basic (no insert), were methylated using *Sss*I methyltransferase (New England Biolabs, Hitchin, UK) according to the manufacturer’s recommendation. In brief, 10–15 µg of plasmid DNA was incubated with or without *Sss*I methyltransferase (20 U/µl; 2 U/µg DNA) in the presence of 640 µM S-Adenosylmethionine (SAM) (New England Biolabs) for four hours at 37 °C, with another 640 µM SAM being added after the first two hours of incubation. Plasmid DNA was purified using QIAquick PCR purification kit (Qiagen). Methylation of plasmid DNA was controlled by digestion using methylation sensitive restriction enzyme *Hpa*II (Thermo Fisher Scientific) for four hours at 37 °C.

### PLIN1 promoter reporter assays

Human mesenchymal stem cells (hMSCs) isolated from adipose tissue and differentiated *in vitro* to adipocytes were used for the transfection with plasmids^42^. Day 9 of differentiated cells were transfected using Neon electroporator (Invitrogen, Carlsbad, CA) according to the manufacturer’s protocol. The cell amount per 10 µl electroporation tip was 100.000 together with 500 ng of methylated or unmethylated pCpGL-basic or pCpGL-PLIN plasmids and 10 ng of a plasmid containing Renilla luciferase gene (Promega, Madison, WI). Electroporation conditions were 1400 Volts, 20 ms width, and 2 pulses. Following electroporation the cells were cultured in 48-well plates for 24 hours there after luciferase activities were measured in cell lysates using Dual Luciferase Reporter Assay System (Promega) according manufacturer’s instructions. Each sample was prepared in quadruplicates and the experiment was repeated three times.

### Global demethylation assays *in vitro*

hMSCs differentiated *in vitro* to adipocytes were treated with 50 µM or 200 µM of DNA methyltransferase inhibitor RG108 (Abcam, Cambridge, UK) at differentiation day 11–12 for 24 h. Medium was collected and the cells were used to isolate RNA/DNA (AllPrep DNA/RNA Kit, Qiagen) and proteins. Isolated DNA was used for measuring global shifts in DNA methylation (Imprint^®^ Methylated DNA Quantification Kit, Sigma Aldrich,) according to the manufacturer’s instructions. Briefly, 60 ng of DNA was diluted in DNA Binding Solution and incubated at 37 °C for 60 mins. After washing, Capture Antibody at dilution 1:1000 was added and incubated at RT for 60 mins. After washing, Detection Antibody was added to each well at dilution 1:1000 and incubated at RT for 30 mins. Thereafter Developing Solution was added and incubated at RT for 1–10 mins. The reaction was stopped by adding Stop Solution and the absorbance was measured at 450 nm (NanoQuant spectrophotometer, Tecan, Männedorf, Switzerland). Cytotoxicity of RG108 was determined in cell culture medium using Cytotoxicity determination kit (Roche Applied Science, Mannheim, Germany) according to the instruction of the manufacturer.

### Analysis of protein expression

Approximately 250,000 hMSCs were lysed in 140 μl RIPA buffer (150 mM sodium chloride, 1% NP-40, 0.5% sodium deoxycholate, 0.1% SDS, 50 mM Tris, pH 8.0). 15–20 µg of total protein was separated by SDS-PAGE and Western blot was performed according to standard procedures. The membranes were blocked in 3% ECL Advance™ Blocking Agent (GE Healthcare, Buckinghamshire, UK). Primary antibodies against Perilipin (GP29, Progen, Heidelberg, Germany) and β-actin (Sigma Aldrich) were used. Secondary guinea-pig/rabbit-IgG antibodies were conjugated to horseradish peroxidase (Sigma-Aldrich). Protein bands were detected by chemiluminescence using ECL™ Select Western Blotting Detection Kit (GE Healthcare) in Chemidoc XRS system (Bio-Rad Larobarotries) and quantified by Quantity One software (Bio-Rad Laboratories).

### Statistical analysis

Statistical analyses were performed in JMP v. 12. Shapiro-Wilks test was used to test whether the distribution of analyzed quantitative variables deviated from normality. Two-group comparisons of normally distributed variables were performed by two-sided Student’s t-test (age, diastolic blood pressure, cholesterol, NEFA, lipolysis measures, adipocyte volume, and *PLIN1* mRNA and protein) and otherwise by Kruskal-Wallis test. Five of nine analyzed CpG-sites were not normally distributed; for simplicity all DNA methylation results were analyzed by Kruskal-Wallis test. Pyrosequencing results were due to small sample size analyzed by non-parametric Kruskal-Wallis test. Relationship between degree of CpG-methylation and quantitative phenotypes was assessed by simple regression. Relationship between *PLIN1* mRNA and quantitative phenotypes was assessed by multiple regression adjusting for batch. Lipolysis values were log_10_-transformed before analysis to become normally distributed. Values are mean + SD.

### Data availability

The datasets generated during and/or analysed during the current study are available from the corresponding author on reasonable request.

## Electronic supplementary material

Supplementary Figure
