# Supplementary material for: Epigenetic Regulation of PLIN1 in Obese Women and its Relation to Lipolysis
Source: Sci Rep. 2017 Aug 31;7:10152. doi: 10.1038/s41598-017-09232-y (PMC5578955; doi:10.1038/s41598-017-09232-y)
Supplement: Supplementary file 1 — Supplementary Figure [file 41598_2017_9232_MOESM1_ESM.pdf]

Epigenetic regulation of *PLIN1* in obese women and its relation to lipolysis

Lucia Bialesova[1], Agné Kulyté[2], Paul Petrus[2], Indranil Sinha[1], Jurga Laurencikiene[2], Chunyan Zhao[1], Karin Dahlman Wright[1], Peter Arner[2], Ingrid Dahlman[2*]

1. Karolinska Institutet, Department of Biosciences and Nutrition, S-141 83 Stockholm, Sweden
2. Karolinska Institutet, Department of Medicine, Huddinge, S-141 86 Stockholm, Sweden

Fig. S1

A

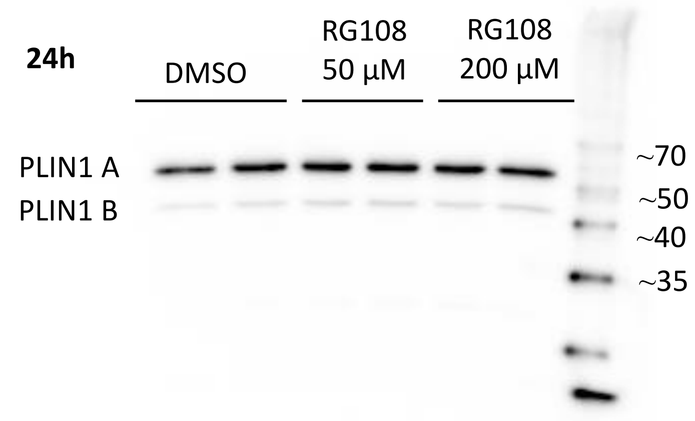

B

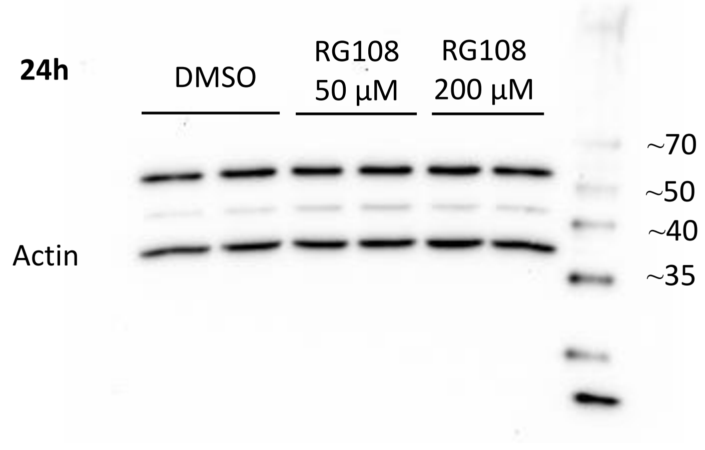

Fig. S1. Full length blot images A. Full length blot image of Figure 4B (PLIN1). Extra protein bands represent PLIN1 expression after 24h treatment with 200 µM RG108. B. Full length blot image of Figure 4B (Actin). Extra protein bands represent Actin expression after 24h treatment with 200 µM RG108.
